# Heterogeneous adaptive behavioral responses may increase epidemic burden

**DOI:** 10.1038/s41598-022-15444-8

**Published:** 2022-07-04

**Authors:** Baltazar Espinoza, Samarth Swarup, Christopher L. Barrett, Madhav Marathe

**Affiliations:** Network Systems Science and Advanced Computing Division, Biocomplexity Institute, University of Virginia, Charlottesville, VA 22904 USA

**Keywords:** Infectious diseases, Behavioural ecology, Infectious diseases, Applied mathematics

## Abstract

Non-pharmaceutical interventions (NPIs) constitute the front-line responses against epidemics. Yet, the interdependence of control measures and individual microeconomics, beliefs, perceptions and health incentives, is not well understood. Epidemics constitute complex adaptive systems where individual behavioral decisions drive and are driven by, among other things, the risk of infection. To study the impact of heterogeneous behavioral responses on the epidemic burden, we formulate a two risk-groups mathematical model that incorporates individual behavioral decisions driven by risk perceptions. Our results show a trade-off between the efforts to avoid infection by the risk-evader population, and the proportion of risk-taker individuals with relaxed infection risk perceptions. We show that, in a structured population, privately computed optimal behavioral responses may lead to an increase in the final size of the epidemic, when compared to the homogeneous behavior scenario. Moreover, we find that uncertain information on the individuals’ true health state may lead to worse epidemic outcomes, ultimately depending on the population’s risk-group composition. Finally, we find there is a set of specific optimal planning horizons minimizing the final epidemic size, which depend on the population structure.

## Introduction

Non-pharmaceutical interventions (NPIs) constitute a suite of front-line behavioral responses whose collective compliance may assemble a *behavioral immune system* at the population scale^1, 2^. Classical mathematical models use a variety of modeling frameworks to study the interplay between disease dynamics and epidemic mitigation policies^3–5^. However, control policies have historically focused on the population-level consequences, neglecting individual-level incentives and costs associated with complying with public health recommendations^6, 7^. Characterization of the effectiveness of control policies and their viability requires understanding how behavioral modifications intended to reshape epidemic dynamics at the population scale interact with individual microeconomics, beliefs, perceptions and health incentives^8–11^. Heterogeneous living conditions (socioeconomic characteristics, beliefs, education, demography, etc.) modulate the behavioral choices individuals make during the epidemic period. Particularly, these impact people’s adherence to recommended control policies^12–14^. Consequently, the extent to which control policies are effective in ameliorating the epidemic burden inherently depends on the affected population’s structure and the associated behavioral responses^15, 16^.

For instance, during the early stage of the COVID-19 pandemic high compliance rates were reported in developed countries^17^. Harper et al. conducted a study about mitigating behaviors during the COVID-19 pandemic in 2020, where about 50% of the participants perceived themselves at “medium” risk of contracting COVID-19, while 33% reported to perceive “low” infection risk^18^. The study by Barber et al.^19^ on assessing COVID-19 perceptions during 2020, reported that 24.5% of participants expressed at least some agreement that people are overreacting. Moreover, Fedele et al. found that vaccine hesitancy in Italy was a major concern with an acceptance of 26.5%^20^. The risk-perception paradigm has historically challenged the effectiveness of population-scale control measures. For example, a study during the 2009 influenza pandemic (pH1N1) in Hong Kong found that 60% of the population perceived low risk of being infected, with a vaccine aversion of around 37% of the population^21^.

Diverse modeling frameworks have been used to characterize the intertwined dynamics generated by the disease dynamics and the set of behavioral responses available to mitigate disease spread^3–5, 22–25^. However, often, the mathematical models do not consider the adaptive nature of the co-evolving epidemic and adaptive behavioral dynamics—in other words, most current behavioral models in epidemiology look only at the current and past system’s state, and use that information to infer individual behavioral responses. Our behavioral model takes into account the behavior of individuals based on their estimate of the epidemic dynamics at the current state and in the future. During an epidemic, the feedback loop between people’s activities to avoid risk of infection and the desire to maintain social interactions and economic productivity creates a complex adaptive system, in which behavioral responses both drive and are driven by the disease transmission process. Recently, the modeling framework proposed by Fenichel et al.^22–25^ envisions individual behavioral adaptations as a Markov decision process by using an epidemiological-economic model. Markov decision processes have found applications and provided foundations in AI for modeling sequential decision-making in diverse contexts^26–28^. In this work we focus on a behavioral aspect extensively documented during the COVID-19 pandemic on different regions, the population’s *heterogeneous behavioral responses* to the risk of infection. We aim to study the impact of heterogeneous behavioral responses on the progression of an epidemic. We extend the epidemiological-economic framework by considering a risk-structured population and information uncertainty. We use a set of ordinary differential equations to model disease progression, and a decentralized Markov decision framework to model the strategic behavior of individuals across risk groups and over different health classes. We assume economic productivity depends on social interactions^29, 30^, and model behavioral changes as adjustments in the contact decisions made by individuals seeking to maximize the net benefits offered by contacts with others, where these also carry a risk of infection. Beyond our explicit formulation of behavioral responses, our results are robust to other types of behavioral responses that ultimately reduce the risk of infection. We incorporate important features of the COVID-19 pandemic: (*i*) a large proportion of infected individuals are asymptomatic or show mild symptoms that allow them to continue social interaction, thus becoming major drivers of transmission^31–33^; (*ii*) the relative infectiousness of exposed and asymptomatic infectious individuals is uncertain^34–36^; and (*iii*) heterogeneous population behavioral responses driven by differential risk perceptions. The role of non-symptomatic (exposed and asymptomatic) but infectious individuals in our behavioral model formulation is critical. Most social interactions require immediate evaluation of the infection risk, which is assumed to be determined by vulnerability cues^37^. Consequently, in the absence of symptoms, exposed and asymptomatic individuals may both behave and be treated by others as if they are uninfected (i.e., in the susceptible state w.r.t. the epidemic dynamics). The potential transmission of COVID-19 during the pre-symptomatic and asymptomatic stages was quickly recognized during the pandemic^38, 39^. Moreover, it is known that non-symptomatic transmission is critical at the early- and long-term epidemic dynamics, so that models not considering this transmission route would exhibit bias on estimates of the basic reproductive number potentially leading to model inaccuracy^40–43^.

We extend the behavioral modeling framework by Fenichel et al.^22–25^ by: (*i*) *coupling a set of Markov decision processes* (MDPs) to incorporate differential adaptive behavioral responses; (*ii*) using a more complex epidemic model of disease transmission; and (*iii*) *including the role of uncertain information* about individual health states, affecting the decision-making processes. Individuals are divided into two classes based on their risk-acceptance behavior. These extensions aim to incorporate pervasive experiences from the ongoing COVID-19 pandemic. Our simulations yield the following insights: (*i*) there is a balance between the reduction of cases due to risk-evaders’ effort to decrease their infection likelihood, and the proportion of the population acting as risk-takers; (*ii*) the risk-reduction and risk-takers trade-off has the potential to increase the attack rate (the proportion of the population infected over the epidemic), and it is sensitive to the proportion of asymptomatic cases and their relative infectiousness; and (*iii*) there is a set of optimal planning horizons that minimize the attack rate. The optimal planning horizons depend on the population’s structure and the group-specific risk sensitivities. Finally, to the best of our knowledge, this is the first time that *multiple MDPs are coupled* to study heterogeneous adaptive behavioral responses in an epidemic model.

## Methods

### Constant contacts model

We assume the affected population is composed of two risk-groups, a fraction *p* of the population is composed of risk-takers (RT) and the remaining fraction $1-p$ are risk-evaders (RE). We differentiate the RT and RE subpopulations by assuming the RE population face a reduced likelihood of infection due to adopting precautionary behaviors. On the other hand, we assume RT do not follow public health recommendations, thus facing a higher risk of infection, relative to the RE population. Political or ideological reasons, economic stress, the lack of reasonable alternatives, epidemic politicization or the lack of trust in public health authorities are some of the documented factors that potentially lead the population to risk the dangers of COVID-19 infection^44, 45^.

**Figure 1:**
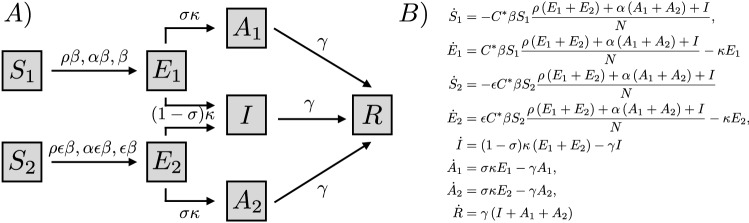
Constant contacts disease model. The affected population is divided into two behavioral groups: risk-takers (RT) and risk evaders (RE), denoted with the subscripts 1 and 2, respectively. Thus, $S_1, E_1,$ and $A_1$ are the susceptible, exposed, and asymptomatic subpopulations of risk-takers, respectively; and similarly $S_2, E_2,$ and $A_2$ for risk evaders. We consider a single symptomatic subpopulation since we assume homogeneous behavior of individuals in this health class.

Previous mathematical models consider complex within-host disease dynamics^46^ or the impact of exogenous factors on the COVID-19 transmission dynamics^47^. In this study, we focus on incorporating individual heterogeneous adaptive behavioral responses, based on group-specific infection risk perceptions. Our model of disease progression assumes that individuals in each behavioral group may show the following health status: Susceptible (*S*), infectious Exposed (*E*), Infectious symptomatic (*I*), infectious Asymptomatic (*A*), and Recovered (*R*). We consider a pre-symptomatic infectious health status (*E*), following evidence suggesting that exposed individuals exhibit a period of viral shedding^38, 48–51^. RT susceptible individuals ($S_1$) can get infected by making contacts with either: symptomatic ones (*I*) with a baseline per-contact likelihood of disease transmission $\beta$, exposed individuals ($E_1$ and $E_2$) with reduced per-contact likelihood of infection $\rho \beta$ , or asymptomatic individuals ($A_1$ and $A_2$) with reduced per-contact likelihood of infection $\alpha \beta$. Similarly RE susceptible individuals ($S_2$) may get infected by making contacts with symptomatic, exposed or asymptomatic individuals at respective likelihoods, $\epsilon \beta$, $\rho \epsilon \beta$, and $\alpha \epsilon \beta$, where $0<\epsilon <1$ corresponds to the reduction of the infection risk given by adopting precautionary behaviors. We assume $C^*$ is the optimal contact rate in the absence of disease transmission, which remains constant in the absence of behavioral adaptations. Due to the absence of adequate data on the specific infectiousness of exposed and asymptomatic COVID-19 infected individuals^49^, we assume these subpopulations are less infectious than symptomatic ones, with $\rho =0.25$, and $\alpha =0.4$. Our model assumes that on average, $1/\kappa$ days after infection, a proportion $\sigma$ of exposed individuals remain asymptomatic, while the rest develop symptoms. Finally, we assume a similar infectious period of $1/\gamma$ days for symptomatic and asymptomatic individuals. Our model for disease progression is sketched in Fig. 1A, and mathematically formalized by the system of ordinary differential equations in Fig. 1B, where ${\dot{f}}$ stands for the time derivative of *f*.

### Heterogeneous adaptive behavior

**Figure 2:**
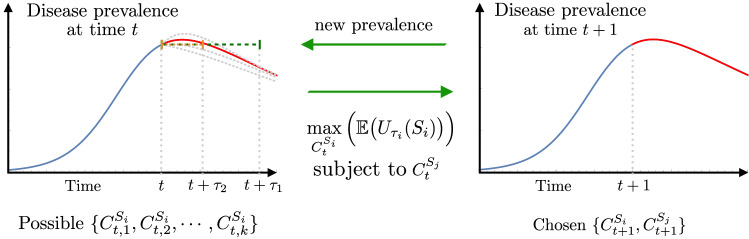
Coupling disease dynamics and forward looking Markov Decision Processes. At each time step the sequential decision process sets a feedback loop between the epidemic state and individual behavioral responses: (*i*) the current disease prevalence defines potential future health state transitions, (*ii*) a projection of the system’s future state over the planning horizon sets the optimization problem, (*iii*) we find the population-specific optimal contact rates that maximize the expected utility of the susceptible population, over the planning horizon and simulate the epidemic model one step forward.

We study the impact of interactions between infected individuals and susceptible individuals dynamically adapting their behavior in response to the perceived risk of infection. Our model focuses on infected individuals with mild or no symptoms and excludes individuals with severe symptoms, since these have minimal interaction with the general population. The economic-epidemiological model we use incorporates the interdependence between the epidemic burden and individual behavioral responses. Figure 2 shows a schematic of the coupling between the mean field epidemiological model and the Markov Decision Processes we use to model heterogeneous adaptive behavioral responses. We formulate a mean-field epidemiological model incorporating explicit contact rates to evaluate the progression of the epidemic, while simultaneously using a Markov Decision Framework to model individual adaptive behavioral responses.

Notice that the optimization processes for RT and RE susceptible individuals are intrinsically coupled. The contact rates selected by a given population affect the overall population’s activity, which in turn impact the population’s mixing. At each time step, we decouple these processes by computing the optimal contact rate at time $t+1$ for a given risk group, assuming the contact rate of the other risk-group to be the same as the one observed at time *t*, the latest sample available. At each time step, the group-specific optimization process incorporates a projection of the system’s future state, by assuming the current prevalence remains constant over the planning horizon. Variations on the future system’s state projection, and on the projection period length, deeply impact the solutions of the optimization problems, consequently impacting the outcomes of the behavioral responses and the epidemic dynamics. By solving the group-specific optimization process at each time-step, we get the privately optimal contact rate for each risk-group. This allows us to simulate the epidemic one step forward and to find the next step disease prevalence level. We iterate this process over the epidemic period to simulate the coevolution of the epidemic process and behavioral adaptations.

The paradigm in our model of human adaptive behavior is given by the trade-off between increasing contacts and the differential risk of infection that these carry for the RT and the RE populations. Individuals in each health class seek to maximize the expected utility due to social interactions, while trying to minimize infection risk, according to the transmission dynamics determined by the constant contact model.

Aside from the conditions that motivate heterogeneous behaviors, in our model individual behavior differs across health classes and risk groups, but individuals with similar health status and within the same risk group are assumed to behave similarly. We may expect some individuals to be satisfied with lower social activity than others however, for simplicity, we assume individual contact preferences have homogeneous and time-invariant structure. That is, individuals similarly value contacts over time, and independently of the epidemic state. Moreover, individual decentralized decisions are assumed to be taken from privately optimal perspectives. The cost-benefit trade-off perceived by individuals does not consider the aggregate effects that changing the pool of contacts has on others’ decisions.

We incorporate heterogeneous behavior in the constant contacts model by weighting the population in each health-class with the corresponding risk-group specific contact rates. Under the adaptive behavior model, the mixing is proportional to the population distribution among health-classes, and conditional on the behaviors determining the dynamic contact rates. Taking the constant contacts model as a baseline, we derive the incidence terms for the RT and RE susceptible individuals by considering the proportion of contacts that a typical individual in the $S_1$ and $S_2$ compartments makes with other infectious individuals,

1 $$\begin{aligned}&\beta C_t^{S_1} S_1 \frac{\rho (C_t^{E_1}E_1+C_t^{E_2}E_2) + \alpha (C_t^{A_1} A_1+ C_t^{A_2} A_2) + C_t^{I} I}{\sum _h C_t^h h}, \text {and}\nonumber \\&\quad \epsilon \beta C_t^{S_2} S_2 \frac{\rho (C_t^{E_1}E_1+C_t^{E_2}E_2) + \alpha (C_t^{A_1} A_1+ C_t^{A_2} A_2) + C_t^{I} I}{\sum _h C_t^h h}, \end{aligned}$$

where $\sum _h C_t^h h$ is the total population activity, for individuals in health classes $h\in \{S_1,S_2,E_1,E_2,I,A_1,A_2,R\}$ selecting contact rates $\{C_t^{S_1},C_t^{S_2},C_t^{E_1},C_t^{E_2},C_t^{I},C_t^{A_1},C_t^{A_2},C_t^R\}$, at time *t*.

Since we assume economic productivity depends exclusively on social interactions, individuals determine the daily optimal contact choices at each time step by maximizing their expected utility $V_t(h)$, depending on their current health status $h\in \{S_1,S_2,E_1,E_2,I,A_1,A_2,R\}$. The health-specific expected utilities $V_t(h)$ comprise the potential benefits obtained by making the optimal contact choice at each future time step during the group-specific planning horizon $\tau _i$. The expected utilities account for potential future transitions to other health states, weighted by the respective transition probabilities, which are given by the system’s current state (the population distribution among health states and their respective contact choices). Individuals evaluate the future benefits/costs assuming the population distribution remains constant during the planning periods. Preferences are assumed single-peaked, so that individuals have a unique optimal contact rate in the absence of disease dynamics. Following the work by Morin et al.^24^, we assume a utility function of the particular form $u(C_t^h)=\left( b C_{t}^{h}-(C_{t}^{h})^ 2\right) ^{\nu }$ , where *b* is the per-day maximum number of contacts possible, $\nu$ is the utility function shape parameter, and $C_{t}^{h}$ is the contact rate of a typical individual with health status *h* at time *t*. We assume individuals within the same risk-group obtain benefits based on a time-invariant utility function shape, regardless of their health status, except symptomatic individuals who get no utility during the infectious period. We use variations of the $\nu$ parameter to modify the marginal benefits of increasing contacts across groups. In other words, our model of heterogeneous behavior assumes individuals across groups show differential disposition to reduce their daily number of contacts. Moreover, we assume risk assessment remains constant over time, therefore the risk-group-specific utility function remains invariant over the epidemic period.

Finally, we incorporate the role of uncertain information on the decision-making process. We let the perceived health status represent a source of information uncertainty, where non-symptomatic individuals (exposed and asymptomatic), unaware of the infection risk they pose to others, may perceive themselves—and be perceived by others—as not presenting a risk of infection^25^.

#### Susceptible, exposed, and asymptomatic individual behavior

We model the susceptible individuals’ daily optimal contact choice problem as a dynamic programming problem, the solution to which generates the privately optimal contact rate^22–25^. Note that, regardless of the behavioral group, individuals follows a *SEIAR* disease progression across health states. Therefore, a single set of Bellman’s equations formulates the optimization problem for both behavioral groups, where behavioral heterogeneity is captured by accordingly changing the health state transition probabilities. Formally, susceptible individuals’ daily optimal contact rate solves the Bellman’s equation,

2 $$\begin{aligned} V_t(S_i)=\max _{C_t^{S_i}}\Big \{u\big (C_t^{S_i}\big )+\delta \big [(1-P^{S_iE_i})V_{t+1}(S_i)+P^{S_iE_i}( V_{t+1}(E_i)) \big ] \Big \}, \end{aligned}$$

where $V_t(S_i)$ is the expected utility of risk group *i* susceptible individuals at time *t*, $V_{t+1}(S_i)$ ($V_{t+1}(E_i)$) is the expected utility of being susceptible (exposed) at time $t+1$, and

3 $$\begin{aligned} P^{S_1E_1}\left( C_t^{S_1}\right) =1-\exp \left( -\beta C_t^{S_1} \frac{\rho (C_t^{E_1}E_1+C_t^{E_2}E_2)+\alpha (C_t^{A_1} A_1+ C_t^{A_2} A_2) + C_t^{I} I}{\sum _h C_t^h h}\right) \end{aligned}$$

is the probability of being infected at time *t* for RT individuals. Since RT and RE individuals have similar disease progressions, Eq. (2) also holds for RE, by adjusting the respective infection risk ($\epsilon \beta$) and the corresponding contact rate ($C_t^{S_2}$), such that

4 $$\begin{aligned} P^{S_2E_2}\left( C_t^{S_2}\right) = 1-\exp \left( -\epsilon \beta C_t^{S_2} \frac{\rho (C_t^{E_1}E_1+C_t^{E_2}E_2)+\alpha (C_t^{A_1} A_1+ C_t^{A_2} A_2) + C_t^{I} I}{\sum _h C_t^h h}\right) . \end{aligned}$$

The optimization problem formalized in Eq. (2) incorporates RT (RE) susceptible individuals’ immediate utility ( $u(C_t^{S_i})$ ), plus the expected future utility discounted at a rate $\delta$. The susceptible individuals’ expected future utility accounts for the expected utility of remaining susceptible at the next time step, $V_{t+1}(S_i)$, with probability $1-P^{S_iE_i}$ and the expected utility of being infected $V_{t+1}(E_i)$ (progressing to the $E_i$ compartment), with probability $P^{S_iE_i}$.

Notice that the solution of the optimization problem for susceptible individuals depends upon the expected utility of exposed individuals. Similarly to Eq. (2), we formulate the Bellman’s equation for exposed and asymptomatic individuals

5 $$\begin{aligned} V_t(E_i) = u(C_t^{S_i})+\delta \big [(1-P^{E_i})V_{t+1}(E_i) + P^{E_i}\big (\sigma V_{t+1}(A_i)+(1-\sigma )V_{t+1}(I)\big ) \big ], \end{aligned}$$

where $P^{E_i}=1-e^{-\kappa }$ stands for the probability of progressing from the $E_i$ health class to either $A_i$ or *I*, with respective probabilities $\sigma$ and $1-\sigma$, and where the expected utility of asymptomatic individuals is given by,

6 $$\begin{aligned} V_{t}(A_i) = u(C_t^{S_i})+\delta \big [(1-P^{A_iR}) V_{t+1}(A_i)+P^{A_iR}V_{t+1}(R)\big ], \end{aligned}$$

with $P^{A_iR}=1-e^{-\gamma }$ representing the probability of recovery.

We assume the absence of symptoms leads exposed and asymptomatic individuals to perceive themselves (and be perceived by others) as susceptible individuals, therefore becoming a source of uncertain information. This is incorporated in their corresponding immediate utilities on Eqs. (5) and (6), by using the term $u(C_t^{S_i})$, where susceptible, exposed, and asymptomatic individuals within the same risk group choose their contact rates in the same way. In other words, we track individual risk-avoidance efforts over health-classes except while infected and recovered.

#### Symptomatic and recovered individual behavior

Since our model for disease progression does not consider potential reinfections, we assume there is no incentive for symptomatic and recovered individuals to adapt their behavior. It follows that symptomatic and recovered individuals make the daily number of contacts that maximizes their net benefits. The expected utility of symptomatic individuals, $V_t(I)$, is given by the Bellman’s equation,

7 $$\begin{aligned} V_{t}(I)=u(C_t^{*})+\delta \big [(1-P^{IR})V_{t+1}(I)+P^{IR}V_{t+1}(R) \big ], \end{aligned}$$

while the expected utility of recovered individuals, $V_t(R)$, is formalized by,

8 $$\begin{aligned} V_{t}(R)=u(C_t^{*})+\delta V_{t+1}(R), \end{aligned}$$

where $P^{IR}=1-\exp ^{-\gamma }$ is the recovery probability.

Note that symptomatic and recovered individual utility expectations represent static problems, since they only depend upon the recovery rate and can be explicitly solved. Although we have not explicitly included a potential contact rate reduction of symptomatic individuals, for instance, due to altruism or sanctions, we can model it by limiting the maximum contacts available for this subpopulation.

## Results

Since the proposed behavior model is not amenable to an analytical solution, we numerically explore the implications of adaptive behavior and uncertain information on the epidemic dynamics. We use the final epidemic size as a metric to assess the impact of behavioral responses on different behavioral and epidemiological scenarios. In the absence of appropriate behavioral data, we assume that individuals make an average of $b=50$ contacts per day. Future utility is discounted at the rate of 5% per year ($\delta =0.99986$), and the utility function parameter value is assumed to be $\nu =0.1$^22, 24^. The discount rate $\delta$ affects the decisions made during the planning horizon by weighting future decisions during the Markov Decision Process. Intuitively, high discount rates bias the decision-making process towards valuing the present more than the future and the potential future consequences. In counterpart, the utility function parameter $\nu$ modulates the immediate utility obtained as well as the benefits (loss) of increasing (reducing) contacts. Intuitively, reducing $\nu$ decreases the utility loss of decreasing contacts, leading to high reductions of the contact rates. In the Supplementary Information, we show that variations on the daily average contacts (*b*), and the discount rate ($\delta$) parameters, modify the sensitivity of the behavioral response, but do not change our qualitative results. Given that the early phase of the epidemic is mainly driven in the absence of behavioral responses, we calibrate the behavior model by making the constant contacts model’s basic reproductive number $({\mathcal {R}}_0)$ to be consistent with early disease dynamics of the COVID-19 pandemic reported in literature. Particularly, since we assumed a totally naïve population, the initial stage of the epidemic is driven in the absence of sanitary recommendations and in the absence of behavioral responses. Therefore, we assumed that at the early phase of the epidemic, the risk of infection is not perceived, and we computed the basic reproductive number assuming a population of risk-takers, ($p=1$). Exposed individuals are assumed to exhibit a 5-day incubation period ($\kappa =1/5$), during which they can transmit the disease with a reduced infectiousness of $\rho =0.25$^38^. Infected individuals recover and cannot infect others on average after 9 days ($\gamma =1/9$) of symptom onset^52^. For our baseline parameters we assume 50% ($\sigma =0.5$) of the infections become asymptomatic^34, 35^, with relative infectiousness of $\alpha =0.4$^53, 54^. These baseline parameters with a per-contact likelihood of infection $\beta =0.01324$ generate a basic reproductive number of 2.4^55, 56^. Finally, we assume *risk-evaders* reduce their infection risk relative to *risk-takers* by adopting safe behaviors, the reduced likelihood of infection of risk-evaders is incorporated by a factor $0\le \epsilon <1$. The set of parameters used in our numerical experiments, unless otherwise indicated, is collected in Table 1.

**Table 1:** Baseline parameters for the constant contacts and adaptive behavior model.

Par.	Description	Value	Ref
$\nu _1$ ($\nu _2$)	Risk-takers (risk-evaders) utility function shape parameter	0.1 (0.05)	^22^
$\tau _1$ ($\tau _2$)	Risk-takers (risk-evaders) planning horizon	14 (14)	Assumed
$\delta$	Discount factor (5% annual rate)	0.99986	^22^
*b*	Maximum number of contacts per day	48	^22^
$\beta$	Likelihood of infection	0.01325	^22, 55, 56^
$\kappa$	Incubation rate	1/5	^38^
$\gamma$	Recovery rate	1/9	^52^
$\rho$	Exposed ind. infectiousness scalar factor	0.25	Assumed
$\alpha$	Asymptomatic ind. infectiousness scalar factor	0.4	^53, 54^
$\varepsilon$	Risk-evaders infectiousness scalar factor	0.7	^53^
$\sigma$	Proportion of asymptomatic ind.	0.5	^34, 35^
*p*	Proportion of risk-taker individuals	Variable	Assumed

### Heterogeneous adaptive responses may increase the attack rate

**Figure 3:**
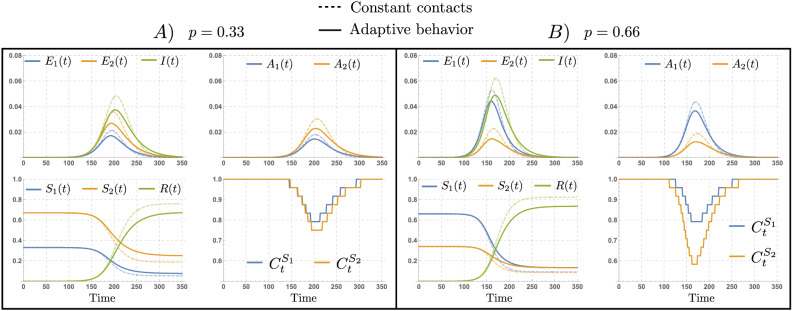
Variations in the proportion of RT individuals impact disease dynamics and behavioral responses. Disease dynamics under constant contacts model (dashed curves) and under adaptive behavior model (thick curves). The scenarios where 33% (**A**) and 66% (**B**) of the population are high-risk takers show distinct disease dynamics, inducing differential behavioral responses ($C^{S_1}_t$ and $C^{S_2}_t$) as a function of the risk perception. The set of parameters used are those in Table 1.

In this section we show there is a trade-off between the reduced infection risk by individuals adopting precautionary measures, i.e., the *risk-evaders* (RE, denoted by $S_2,E_2$ and $A_2$), and the non-compliant individuals, whom we refer to as the *risk-takers* (RT, denoted by $S_1,E_1$ and $A_1$). Moreover, we show that this trade-off is dependent on the proportion of asymptomatic cases. In Fig. 3 we show selected simulations of the disease dynamics for the constant contacts model (dashed), and for the adaptive behavior model (solid), assuming a scenario where RE reduce their infection risk by $30\%$ ($\epsilon =0.7$). In panel (A) we assume 33% of the population are RT ($p=0.33$), while in panel (B) we assume 66% of the population are RT ($p=0.66$). As expected, our simulations in panel (A) show that the epidemic is mainly driven by infections in the RE population. Interestingly, despite the high prevalence levels in the RE population, the behavioral responses of the RT and the RE populations produced in this scenario are similar. Both populations reduce their respective contact rates, $C_t^{S_1}$ and $C_t^{S_2}$, by around 20% during the peak time. On the other hand, panel (B) shows a faster and earlier epidemic with a higher peak, mainly driven by infections in the RT population. In this scenario, the behavioral responses produced diverge significantly. While RT individuals reduce their contacts by 20% during the peak time (similar to the scenario in panel (A)), RE individuals reduce their contacts by around 40% during the peak time.

The scenarios chosen assume highly distinct population structures in terms of the risk-taker and risk-evader populations ($p=0.33$ and $p=0.66$), which in turn produce contrasting disease dynamics (propagation speed and peak size), ultimately inducing different behavioral responses among the $S_1$ and $S_2$ populations.

Figure 3 show that while both scenarios exhibit reduction of contacts due to behavioral adaptations to avoid infection risk, the level of contact reduction is sensitive to the population’s composition, since this impacts the prevalence levels attained. Moreover, our simulations suggest that the higher the prevalence levels reached—given by predominance of the risk-taker population—the stronger the behavioral response of the risk-evader population.

**Figure 4:**
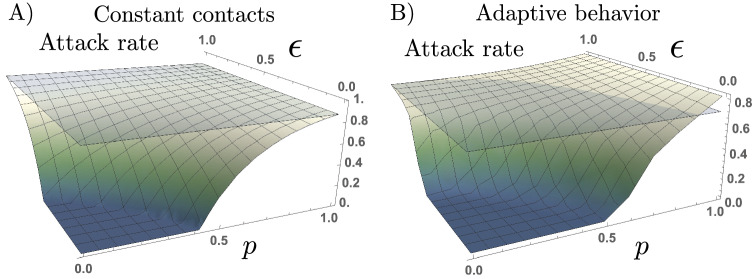
Risk-takers may increase the attack rate under adaptive behavioral response with uncertain information. Attack rate as a function of the proportion of both high-risk takers (*p*) and reduced risk of infection of the risk-evaders subpopulation ($\varepsilon$). The set of parameters used are those in Table 1.

Intuitively, the extent to which efforts from the risk-evaders effectively reduce the epidemic burden depends on the proportion of risk-takers in the population. We now explore the trade-off between the risk reduction by RE individuals ($\varepsilon$), and the proportion of risk-takers (*p*). Notice that this trade-off is shown only on the attack rate produced for the behavioral response model. Figure 4 shows that, for the constant contacts model, the attack rate is always below the baseline scenario $(p=0,\varepsilon =1)$, monotonically decreasing as either $\varepsilon$ or *p* decreases. This is an expected result, since the homogeneous assumption of the constant contacts model results in similarly weighting the potential risk reduction by RE individuals, and the increased infection risk that RT individuals pose to others.

However, by modeling the group-specific heterogeneous behavioral responses, we capture novel insights on the impact of RT’s infectiousness and adaptive contact rates. For the adaptive behavior model, the attack rate attained in the presence of risk-takers may overcome the reduction of cases due to the RE individuals efforts, which ultimately takes the attack rate above the baseline scenario. In other words, there is trade-off between the increased expected activity and the infection risk of RT individuals that balance the reduction of cases due to RE efforts. Particularly, the trade-off shown on the attack rate is a function of the proportion of RT in the population (*p*), and the RE infection risk reduction ($\varepsilon$).

### Risk misperception modulates the trade-off between risk-takers and risk-evaders

The role of asymptomatic individuals on the spread of COVID-19 has been mostly studied from the perspective of the silent infections produced^31–33^. Recently, Espinoza et al. studied the potential impact that behavioral responses based on infection risk misperceptions posed by asymptomatic individuals produce on the epidemic burden^25^. Here, we focus on the role of asymptomatic cases on modulating the trade-off produced by the RE and RT populations. Particularly, we focus on how the presence of asymptomatic cases leading to risk misperceptions exacerbate the impact of RT individuals.

**Figure 5:**
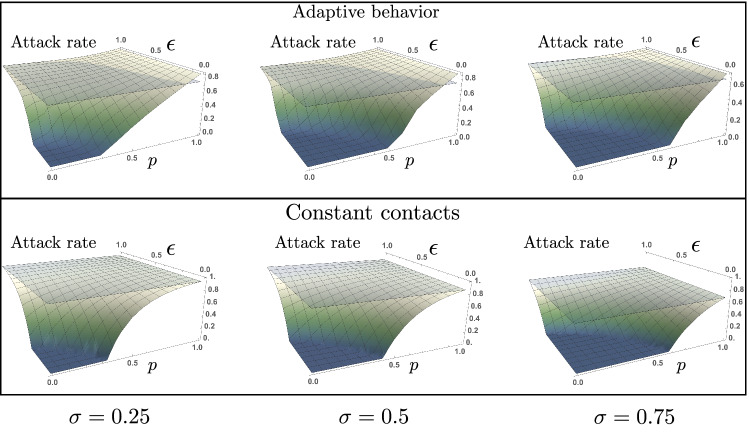
The trade-off between RE’s risk-reduction and the proportion of RT individuals depends on the proportion of asymptomatic cases. Attack rates obtained with the constant contacts model, and with the adaptive behavior model, for the scenarios where 25%, 50%, or 75% of infections are asymptomatic. The $(p, \varepsilon )$ regions where the attack rate is increased compared to the baseline scenario ($p=0, \varepsilon =1$) is modulated by the presence of asymptomatic cases. The set of parameters used are those in Table 1.

Given that the asymptomatic/symptomatic ratio, and the potential infectiousness of COVID-19 asymptomatic individuals is uncertain, we explore the impact that the proportion of asymptomatic cases has on the trade-off between the RE infection risk reduction and the proportion of RT in the population. Figure 5 exhibits the attack rates obtained by assuming different proportions of asymptomatic cases ($\sigma =0.25,0.5$ and $\sigma =0.75$), for the different scenarios of RE efforts ($\varepsilon$), and for varying proportions of RT individuals (*p*).

We focus on the impact that varying the proportion of asymptomatic cases has on the $(p,\varepsilon )$ trade-off exhibited in the attack rate. In general, the lower the proportion of asymptomatic cases, the higher the attack levels attained. However, the lower the proportion of asymptomatic cases, the lower the proportion of RT individuals required to take the attack rate above the baseline level.

Our results highlight the importance of addressing differential behavioral responses during an epidemic. We show that in a population composed of two risk-groups, the consequences of differential adaptive behavioral responses can be characterized by the trade-off between the efforts to reduce infection risk and the proportion of the population not following precautionary behaviors. Moreover, our results highlight that risk-taker individuals play a dual role in the epidemic burden: besides the higher risk of infection these individuals pose to others, the high contact rates associated to this population may produce more cases than that expected using the analogous constant contacts model.

### Disease reporting drives behavioral responses strengths

**Figure 6:**
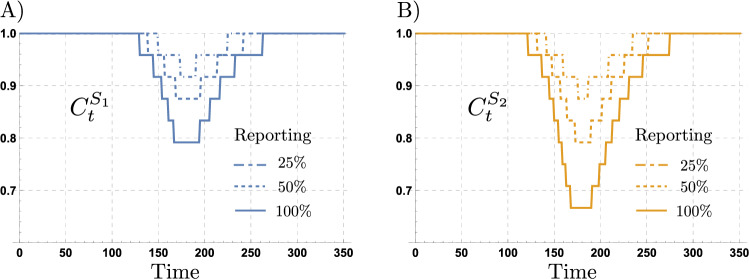
Risk-group contact rates for different reporting levels. Risk-taker and risk-evader contact rates for levels of reporting of $25\%$, $50\%$ and $100\%$. The risk-evaders’ contact rate is more sensitive to changes in the reporting levels, compared to risk-takers’ contact rate. The set of parameters used are those in Table 1.

We study the impact that differential testing capacity has on the perceived infection risk and on the behavioral responses produced. Similar to other epidemic models including human behavioral responses^3, 57^, our previous simulations assume a framework of complete information, however this may not be achievable for large epidemics. In reality, the risk perceptions during an epidemic, and consequently the induced behavioral responses, depend upon the region-specific surveillance efforts. We explore the impact of distinct surveillance regimes by varying the level of disease prevalence observed through testing. This is incorporated in our behavior model, by including the scalar factor $0\le \varphi \le 1$ in the incidence terms of Eq. (1). In this way, the surveillance level modifies the perceived probability of infection in the Bellman’s equations by only perceiving a fraction of the infectious population, ultimately affecting the behavioral decision chosen. In most countries, reporting is biased towards the identification of symptomatic individuals^58^. For simplicity, in our model we assume reporting impacts the perceived infection risk by reducing the overall perceived disease prevalence level. We incorporate a scalar factor that modulates the perceived infectious subpopulations (exposed, symptomatic and asymptomatic), proportional to its size. In other words, we do not incorporate the bias produced by mostly reporting symptomatic individuals. Figure 6 shows the impact of different levels of reporting on the contact rates of the risk-takers and risk-evaders. Our simulations show that behavioral responses of both risk-groups are highly sensitive to changes in reporting levels. Moreover, risk-evaders’ behavioral responses dramatically reduce as the level of reporting decreases.

**Figure 7:**
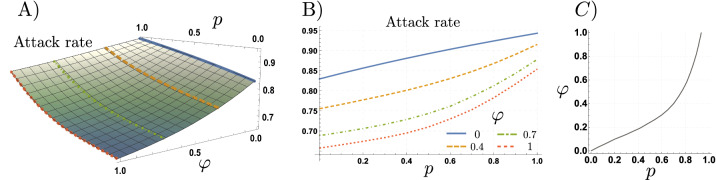
Attack rate for different reporting levels. (**A**) shows the attack rate as a function of the reporting level ($\varphi$), and the proportion of the population behaving as risk-takers (*p*). (**B**) shows the non-linear effect of increasing the proportion of risk takers in the population, for different values of reporting. (**C**) shows the trade-off between the proportion of risk-takers and the surveillance effort $(p,\varphi )$, such that the attack rate attained in the scenario of no surveillance and no risk-takers $\big ((p,\varphi )=(0,0)\big )$, remains constant. The set of parameters used are those in Table 1.

Our next simulations explore the effects of different reporting levels ($\varphi$) on the attack rate attained for the adaptive behavior model, for populations composed of different proportions of risk-takers (*p*). Figure 7 shows that for the adaptive behavior model, the impact of risk-takers on the attack rate depends upon the reporting levels. Particularly, the extent to which behavioral responses decrease the attack rate depends on the reporting levels. For high reporting levels ($\varphi \approx 1$), the attack rate shows a concave up shape to increments in the proportion of risk-takers. In counterpart, for low reporting levels, the attack rate shows a concave down shape to increments in the proportion of risk-takers.

Particularly, Fig. 7B shows that, as expected, the higher the reporting level $(\varphi )$, the lower the attack rate attained for similar population structures (corresponding values of *p*). Moreover, the concavity of the attack rate curves with reporting ($\varphi >0$), suggest that reporting increases population’s robustness to increments of the risk-takers population. Figure 7C depicts the trade-off between reporting and the proportion of risk-takers in the population, taking as a reference the attack rate attained for a population with no risk-takers and no reporting ($\varphi =p=0$). Interestingly, in populations mainly composed by risk-evaders ($p\approx 0$), low reporting levels ($\varphi \approx 0$) overcome the impact of risk-takers. However, for the scenario of a population with majority of risk-takers, very high reporting levels are needed to mitigate the impact of risky behavior on the attack rate. The important result here is that, in the presence of behavioral responses low testing levels may be compensated by a population adopting safe behaviors. On the other hand, high reporting levels may compensate risky behaviors in a population mainly composed by risk-takers.

Our simulations help us to get insight on the weakening effect of under-reporting on the adaptive behavioral responses based on risk perception. Moreover, our results shed light on the disease dynamics produced by NPIs aimed to modify individuals behavior, and the role of surveillance efforts. Given that only a fraction of the epidemic is perceived through testing, the impact of risk misperception modulating individual behavioral responses increases as reporting decreases. In this scenario, risk misperception not only arises due to pre-symptomatic and asymptomatic individuals, but also due to the region-specific testing limitations. It follows that low reporting levels in regions with very limited testing rates, would lead to weak behavioral responses even if the population is mainly composed of risk-evaders.

### Optimal planning horizons minimizing the attack rate

While the heterogeneous living conditions can modulate behavioral choices leading individuals to adopt high- or low-risk behaviors, these also may impose limitations on the way individuals plan ahead and seek for the optimal future behavior. Our model of behavioral responses assumes each risk-group seeks for the contact rates that maximize their expected utility over independent planning horizons. While both optimization processes are intrinsically connected, we let each risk-group assess the potential future outcomes based on its own planning horizon. In this section we explore the impact that the planning horizons of the two risk groups, $\tau _1$ and $\tau _2$, have on the attack rate.

**Figure 8:**
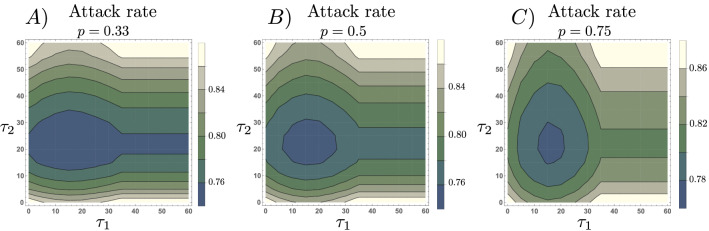
There is a set of planning horizons minimizing the attack rate. The figure shows attack rate level curves as a function of the risk-takers and risk-evaders planning horizons, $\tau _1$ and $\tau _2$, respectively. Differences in group-specific infection risks ($\beta$ and $\epsilon \beta$) and group-specific risk assessment modify the optimal planning horizon. The set of parameters used are those in Table 1.

In Fig. 8, we show selected scenarios of the attack rate for the adaptive behavior model, as a function of the RT planning horizon ($\tau _1$), and the RE planning horizon ($\tau _2$). We consider three scenarios: where the population is mostly composed by RE ($p=0.33$), where the population is balanced with both risk-groups ($p=0.5$), and where most of the population are RT ($p=0.75$). Our simulations show that depending on the population structure in terms of the RE/RT, there exists a combination of planning horizons that minimizes the attack rate. Moreover, the selected simulations show that the impact of varying the RT and the RE planning horizons is not symmetric, regardless of the RT/RE proportions. This is an expected result, given the different behavioral responses across risk-groups due to distinct infection risk perceptions.

Moreover, consistent with our simulations in Fig. 3, the attack rate under adaptive behavioral responses is more sensitive to changes in the planning horizon of RE individuals than for RT ones. In other words, unless the population is mainly composed of RT individuals ($p=0.75$), increments in the planning horizon of the RE individuals ($\tau _2$) impact the attack rate more than the corresponding increment of the RT population’s planning horizon ($\tau _1$).

## Discussion

We focus on a behavioral aspect extensively documented during the COVID-19 pandemic, the population’s *heterogeneous behavioral responses*. While the effectiveness of non-pharmaceutical interventions (NPIs) has been thoroughly studied^12, 59^, behavioral polarization and heterogeneous adherence to recommended policies have been widely reported^60, 61^. Political or ideological reasons, economic stress, or the lack of reasonable alternatives are aspects leading people to maintain social interactions despite the risk of COVID-19 infection^45, 62^. The evidence to date shows that behavioral polarization is pervasive worldwide. The multiple aspects reducing NPIs compliance seem to be reinforced in scenarios where mandates rely on a decentralized governance system. For instance, before vaccines were widely available in the U.S., mask mandates were independently and unevenly enforced at different levels across counties^63^. Despite public health experts endorsed the effectiveness of mask wearing in multiple times, mask wearing mandates along with the effectiveness of masks were the center of national debate^64^. Another example is vaccine hesitancy, which has a long history of being an enormous challenge to control diseases worldwide^65^. Even though multiple vaccines against SARS-CoV-2 are currently available, mostly in developed countries, vaccine hesitancy continues to be a challenge in the containment of the ongoing COVID-19 pandemic^66^. Compliance to social distancing mandates has also shown polarized responses. Multiple social aspects like economic stress, epidemic politicization, or lack of trust in public health authorities have been reported as factors that reduce social distancing adherence^67^.

To reveal the importance of heterogeneous behavioral responses, we study the co-evolving dynamics of heterogeneous adaptive human behavior and disease transmission. We find that adaptive behavioral responses produced by the perceived risk of infection dynamically modify the group-specific contact rate, which modulates the epidemic dynamics. Our results exhibit a trade-off between the efforts to avoid infection by the risk-evader population, and the proportion of risk-taker individuals with relaxed infection risk perceptions. This trade-off defines a threshold in terms of the final epidemic size. Heterogeneous behavioral responses in a structured population may increase or reduce the final epidemic size, relative to final size attained by the analogous population exhibiting homogeneous behavioral responses. In other words, in a structured population, the privately optimal behavioral responses may lead to an increase in the final size of the epidemic.

On the other hand, our simulations show potential impacts of biased adaptive behavioral responses, for instance, due to uncertainty about the true health status of individuals. Given that most social interactions are subject to immediate evaluation of the infection risks, individuals respond to easily observable cues—specifically the presence or the absence of symptoms^68^. It follows that understanding of infection risk is assumed to be determined by vulnerability cues^37^ and the absence of these signals would lead to, at best, a weak behavioral response to individuals exhibiting mild or no symptoms. The impact of risk misperception on the contact rate due to the perceived lack of symptoms in the asymptomatic population has the potential to balance or to be surpassed by the increasing contact rates of asymptomatic individuals, producing more secondary infections. Our simulations show that group-specific optimal behavioral responses and planning horizons may differ among individuals, depending on the associated risk of infection. We found that risk assessment, information accuracy, and willingness to follow precautionary behaviors markedly impact epidemic outcomes. We found the set of optimal planning horizons to be dependent on the population structure in terms of risk-takers and risk-evaders individuals.

We let differential risk perceptions induce heterogeneous adaptive behavioral responses among risk-taker and risk-evader individuals. To model the independently chosen but interconnected behavioral responses, our model of adaptive behavior couples a set of Markov decision processes, formalized via Bellman’s equations. Our model of adaptive behavioral response is based on a projection of the system’s future state up to a specific planning horizon. Individuals seek to balance the cost and benefits of social interactions over the planning horizon, subject to their group-specific risk perception. To model individual adaptive behavior during an ongoing epidemic, we use a decision-making framework that incorporates the private benefits and costs of social interactions. Individuals are assumed to be naive to the impact their decisions impose on others, they do not realize the external costs and benefits of their behavior. Consequently, the role of empathy or social group affinities are not incorporated in the decision-making process. Another critical aspect of the behavioral model is the symmetric, uni-dimensional and single peaked utility function, widely used in economic theory^69^, which allows us to focus on the costs and benefits of social interaction decisions while defining a single preferred contact rate. We model optimal decision-making solely as a function of infection risk aversion. We recognize that many other factors influence the decision-making process in the ongoing pandemic, where these may be characterized as the balance of independent risks, accumulated knowledge and projected benefits^11, 70^. Politicization of the epidemic and the limited capacity to respond by those with low incomes is partially reflected in the population’s structured behavioral responses. Nonetheless, we aim to get insights on the impact that heterogeneous adaptive human behavior produces on the system dynamics by using a parsimonious mechanistic model. Thus, effective control measures intended to induce behavioral responses should address the potential heterogeneous compliance levels and their implications. For instance, the trade-off between reducing risk efforts and the proportion of the population following risky behaviors may be adjusted by enforcing individual-level incentives.

## Conclusions

In this paper, we studied the impact of heterogeneous adaptive human behavior on epidemics from the perspective of complex adaptive systems. That is, in our model, the individual behavioral responses and the epidemic dynamics are intertwined. The changing risk of infection drives behavioral adaptations, which in turn modulates the epidemic evolution. Besides the differential behavioral response induced by individuals’ health status, we assume heterogeneous living conditions lead to differential risk perceptions that ultimately modulate behavioral choices among the risk-groups populations. We assume the population under study is structured by two risk-groups, exhibiting differential sensitivity to the changing infection risk. The paradigm in our model of human adaptive behavior is given by the trade-off between increasing contacts and the differential risk of infection that these carry for the subpopulations.

Our results suggest that in a population consisting of two risk-groups, the extent to which adaptive behavior ameliorates the epidemic burden, depends upon the proportion of the population highly sensitive to the infection risk. Moreover, disparities between risk perception among risk-groups lead to larger epidemics, compared to the ones obtained when risk sensitivity is homogeneous in the population. This highlights the complex underlying mechanisms that lead to highly diverse epidemiological outcomes in regions exhibiting dramatic differences in living conditions. The impact of available information on the behavioral responses was also considered. By assuming different surveillance scenarios, we found that reporting plays a key role on promoting timely and accurate behavioral responses. Moreover, the population structure poses a trade-off between the reporting levels and the proportion of the population sensitive to the infection risk. In other words, similar epidemic sizes can be attained in both scenarios: (*i*) where the majority of the population is risk sensitive, but the surveillance level is low, and (*ii*) where the majority of the population is not risk sensitive and the surveillance level is high. Our work show epidemiological consequences of heterogeneous adaptive human behavior in terms of the expected final epidemic size. However, we also emphasize the importance of addressing population heterogeneity driving differential behavioral responses and the emergent trade-offs. Finally, we show that sources of misinformation can lead to inaccurate assessment of the epidemiological landscape, thus counteracting the benefits of behavioral adaptations.

We conclude with a few directions for future work. A first obvious extension is to consider additional groups. Second, extension of the model to represent adaptive behavior during various phases of an epidemic is of interest—in other words, the notion of risk perception is not held constant throughout the epidemic. This can of course be done computationally, but needs to be based on social and behavioral theories. Third, extending the model to networked systems would be interesting but poses new computational challenges. In a networked system, each individual can plan and adapt based on the *local information*. But computationally, this becomes challenging as adaptive behavior now needs to be computed on a per node basis.

## Supplementary Information

Supplementary Information.
