# Supplementary material for: Heterogeneous adaptive behavioral responses may increase epidemic burden
Source: Sci Rep. 2022 Jul 4;12:11276. doi: 10.1038/s41598-022-15444-8 (PMC9252562; doi:10.1038/s41598-022-15444-8)
Supplement: Supplementary file 1 — Supplementary Information. [file 41598_2022_15444_MOESM1_ESM.pdf]

Supplementary Material
Heterogeneous Adaptive Behavioral Responses May Increase Epidemic Burden

Baltazar Espinoza, Samarth Swarup, Christopher L. Barrett, Madhav Marathe

A The basic reproductive number and final epidemic size for the constant contacts model

Based on the constant contacts model detailed in the main manuscript, and formalized by the following set of ODEs

$$\begin{aligned}
\dot{S}_1 &= -C^*\beta S_1 \frac{\rho\left(E_1+E_2\right)+\alpha\left(A_1+A_2\right)+I}{N}, \\
\dot{E}_1 &= C^*\beta S_1 \frac{\rho\left(E_1+E_2\right)+\alpha\left(A_1+A_2\right)+I}{N} - \kappa E_1, \\
\dot{S}_2 &= -\epsilon C^*\beta S_2 \frac{\rho\left(E_1+E_2\right)+\alpha\left(A_1+A_2\right)+I}{N}, \\
\dot{E}_2 &= \epsilon C^*\beta S_2 \frac{\rho\left(E_1+E_2\right)+\alpha\left(A_1+A_2\right)+I}{N} - \kappa E_2, \\
\dot{I} &= (1-\sigma)\kappa\left(E_1+E_2\right) - \gamma I, \\
\dot{A}_1 &= \sigma\kappa E_1 - \gamma A_1, \\
\dot{A}_2 &= \sigma\kappa E_2 - \gamma A_2, \\
\dot{R} &= \gamma\left(I + A_1 + A_2\right),
\end{aligned} \tag{A.1}$$

we use the next generation approach [1, 2], to compute model (A.1) basic reproductive number. We consider the infectious compartments E_1, E_2, I, A_1, A_2, and define

$$\mathcal{F} = \begin{pmatrix} C^*\beta S_1 \frac{\rho(E_1+E_2)+\alpha(A_1+A_2)+I}{N} \\ \epsilon C^*\beta S_2 \frac{\rho(E_1+E_2)+\alpha(A_1+A_2)+I}{N} \\ 0 \\ 0 \\ 0 \end{pmatrix} \quad \text{and} \quad \mathcal{V} = \begin{pmatrix} \kappa E_1 \\ \kappa E_2 \\ \gamma I - (1-\sigma)\kappa(E_1+E_2) \\ \gamma A_1 - \sigma\kappa E_1 \\ \gamma A_2 - \sigma\kappa E_2 \end{pmatrix},$$

where the next generation matrix is given by $FV^{-1}|_{\{S_1=pN, S_2=(1-p)N\}}$ and the basic reproductive number given by its spectral radius

$$\mathcal{R}_0 = (p+(1-p)\epsilon)C^*\beta\left(\frac{1-\sigma}{\gamma}+\frac{\alpha\sigma}{\gamma}+\frac{\rho}{\kappa}\right), \tag{A.2}$$

with F and V representing the Jacobian matrices of $\mathcal{F}$ and ν, respectively. The basic reproductive number (A.2) accounts for the average secondary infections produced by risk-taker and risk-evader individuals. Notice that in the absence of compliant individuals ($p = 1$), the basic reproductive number incorporates the secondary cases produced by infectious exposed individuals ($\rho C^*\beta/\kappa$), asymptomatic infectious individuals ($\sigma\alpha C^*\beta/\gamma$), and symptomatic individuals ($(1-\sigma)C^*\beta/\gamma$)

$$\mathcal{R}_0 = C^*\beta\left(\frac{1-\sigma}{\gamma}+\frac{\sigma\alpha}{\gamma}+\frac{\rho}{\kappa}\right). \tag{A.3}$$

During the early stage of an epidemic, the disease propagates mainly in the absence of sanitary recommendations, on a population almost completely susceptible. Therefore, in the absence of human behavioral responses, the potential of an epidemic to propagate among the population is captured by its basic reproductive number. We use Expression (A.2) to explore the trade-off between the proportion of risk-takers (p) and the risk-evaders'

efforts to reduce their infection risk (ϵ), that makes an epidemic mathematically sustainable ($\mathcal{R}_0 > 1$), in the absence of behavioral responses.

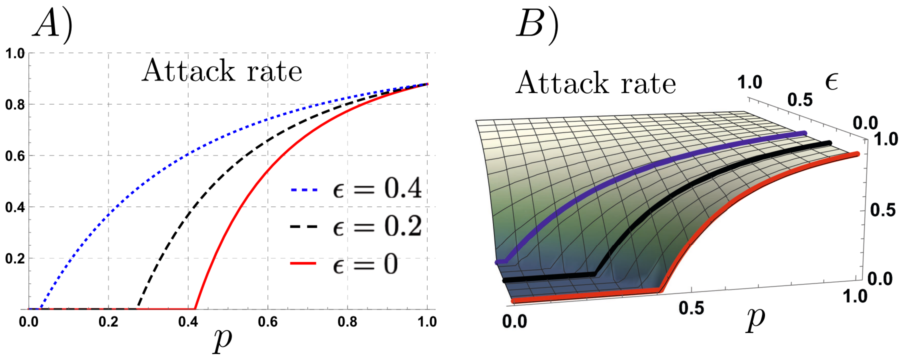

Figure A.1: **The trade-off between the proportion of risk-takers (p) and the relative infectiousness of risk-evaders (ϵ), on the attack rate**. The selected simulations show that a COVID-19-like epidemic ($\mathcal{R}_0 = 2.4$), propagating in the absence of people adopting precautionary behaviors, is mathematically sustainable whenever the proportion of risk-takers is greater than 40% of the population, regardless of the risk-evader containment efforts ($\epsilon > 0$). Parameter values: $\gamma = \frac{1}{9}, \kappa = \frac{1}{5}, \beta = 0.01325, \rho = 0.25, C^* = 24$.

Figure A.1 shows that in a polarized population, a COVID-19-like epidemic is mathematically sustainable if at least 40% of the population do not comply with public health recommendations. Moreover, our simulations show that in the presence of individuals following public health recommendations ($0 < p < 1$), the proportion of risk-taker individuals producing a sustainable epidemic decreases as a function of the risk-evaders' efforts to reduce their infectious risk.

B Coupling the epidemic model and Markov Decision Processes

We formulate a mean-field model of disease progression that explicitly incorporates the population-specific and time-dependent contact rates. For individuals in health classes $h \in \{S_1, S_2, E_1, E_2, I, A_1, A_2, R\}$ we assume respective contact rates $\{C_t^{S_1}, C_t^{S_2}, C_t^{E_1}, C_t^{E_2}, C_t^{I}, C_t^{A_1}, C_t^{A_2}, C_t^{R}\}$, at time t. The goal of the model is to find the optimal contact rates $C_t^{S_1}$ and $C_t^{S_2}$, at each time step over the epidemic period. Following our assumption of information uncertainty among non-symptomatic individuals E_i and A_i, we assume the optimization process of these health classes follows the corresponding contact rates of susceptible individuals in the same risk-group.

We initialize the epidemic model by letting all populations make the contact rates that maximize immediate utility C^*, the optimal contact rate at the disease-free equilibrium. At each time step, the disease prevalence defines the probabilities of infection, subject to the risk-group contact rates $P^{S_1E_1}(C_t^{S_1})$ and $P^{S_2E_2}(C_t^{S_2})$, for risk-takers and risk-evaders, respectively. The per-capita transition rates to the exposed compartment, for the risk-specific susceptible individuals, are given by the respective incidence terms,

$$\lambda_{S_1} = -\beta C_t^{S_1} \frac{\rho(C_t^{E_1}E_1 + C_t^{E_2}E_2) + \alpha(C_t^{A_1}A_1 + C_t^{A_2}A_2) + C_t^I I}{\sum_h C_t^h h}, \text{and} \tag{B.1}$$

$$\lambda_{S_2} = -\epsilon\beta C_t^{S_2} \frac{\rho(C_t^{E_1}E_1 + C_t^{E_2}E_2) + \alpha(C_t^{A_1}A_1 + C_t^{A_2}A_2) + C_t^I I}{\sum_h C_t^h h}, \tag{B.2}$$

where, for instance, the infection force that RT susceptible individuals face at each time step is given by,

$$-\beta \underbrace{C_t^{S_1}}_{\substack{\text{RT susceptible ind.} \\ \text{contact rate}}} \left(\rho \underbrace{\frac{(C_t^{E_1}E_1 + C_t^{E_2}E_2)}{\sum_h C_t^h h}}_{\substack{\text{Prop. of contacts made} \\ \text{with exposed ind.}}} + \alpha \underbrace{\frac{(C_t^{A_1}A_1 + C_t^{A_2}A_2)}{\sum_h C_t^h h}}_{\substack{\text{Prop. of contacts made} \\ \text{with asymptomatic ind.}}} + \underbrace{\frac{C_t^I I}{\sum_h C_t^h h}}_{\substack{\text{Prop. of contacts made} \\ \text{with symptomatic ind.}}} \right), \tag{B.3}$$

and similarly for RE individuals.

Finally, the exponential shape of the transition probability is a property inherited by the ODE model formulation. The infection process for susceptible risk-taker and risk-evader individuals can be modeled as

$$\dot{S}_1 = -P^{S_1E_1}(C_t^{S_1})S_1, \tag{B.4}$$

$$\dot{S}_2 = -P^{S_2E_2}(C_t^{S_2})S_2, \tag{B.5}$$

where $S_1(0) = pN$ and $S_2(0) = (1-p)N$. Hence, $S_1(t)/pN = e^{-P^{S_1E_1}(C_t^{S_1})t}$, where $e^{-P^{S_1E_1}(C_t^{S_1})t}$ denotes the proportion of risk-taker susceptible individuals at time $t = 0$, who remain susceptible at time $t = t$. It follows that the probability a susceptible risk-taker (risk-evader) individual gets infected at time t is respectively given by,

$$P^{S_1E_1}(C_t^{S_1}) = 1 - \exp\left(-\beta C_t^{S_1} \frac{\rho(C_t^{E_1}E_1 + C_t^{E_2}E_2) + \alpha(C_t^{A_1}A_1 + C_t^{A_2}A_2) + C_t^I I}{\sum_h C_t^h h}\right), \tag{B.6}$$

$$P^{S_2E_2}(C_t^{S_2}) = 1 - \exp\left(-\epsilon\beta C_t^{S_2} \frac{\rho(C_t^{E_1}E_1 + C_t^{E_2}E_2) + \alpha(C_t^{A_1}A_1 + C_t^{A_2}A_2) + C_t^I I}{\sum_h C_t^h h}\right). \tag{B.7}$$

C Adaptive human behavior using Bellman's equations

In this section we describe the formulation of the set of Bellman's equations that formalize the optimization process for each risk group in our adaptive behavior model. The state transitions needed to formulate the set of Bellman's equations are given by the potential future health state transitions defined by our model of disease progression. Particularly, in the present work we assume a group-specific SEIAR-like model. The daily optimal contact rate for susceptible individuals solves the Bellman's equation,

$$V_t(S_i) = \max_{C_t^{S_i}}\{ \underbrace{u(C_t^{S_i})}_{\text{Immediate utility}} + \delta \underbrace{[(1 - P^{S_iE_i})V_{t+1}(S_i) + P^{S_iE_i}V_{t+1}(E_i)]}_{\text{Future expected utility}}\}, \tag{C.1}$$

that is, the optimal contact rate for susceptible individuals at time t is given by the contact rate that maximized the expected utility over the planning horizon τ_i. The expected utility is composed of the immediate utility, $u(S_i, C_t^{S_i})$, generated by making $C_t^{S_i}$ contacts at the current time and the discounted (with a discount factor δ) expected future utility, as follows:

$$\underbrace{(1 - P^{S_iE_i})}_{\substack{\text{Prob. of remaining susceptible} \\ \text{in group } i \text{ at time } t+1}} \underbrace{V_{t+1}(S_i)}_{\substack{\text{Expected utility being} \\ \text{susceptible at time } t+1}} + \underbrace{P^{S_iE_i}}_{\text{Prob. of getting infected}} \underbrace{V_{t+1}(E_i)}_{\substack{\text{Expected utility being} \\ \text{exposed at time } t+1}}. \tag{C.2}$$

The solution of the Bellman's equation for susceptible individuals requires to compute the expected utility of susceptible individuals conditional on a potential future transition to the exposed compartment $V_{t+1}(E_i)$.

The expected utility of exposed individuals is given by the following Bellman's equation that accounts for the immediate and expected future utility over the planning horizon.

$$V_t(E_i) = \underbrace{u(C_t^{S_i})}_{\text{Immediate utility}} + \delta \underbrace{[(1 - P^{E_i})V_{t+1}(E_i) + P^{E_i}(\sigma V_{t+1}(A_i) + (1-\sigma)V_{t+1}(I))]}_{\text{Future expected utility}} \tag{C.3}$$

Following our information uncertainty assumption, we assume exposed individuals mimic susceptible ones. Consequently, their immediate utility is computed using the contact rate corresponding to susceptible individuals in the same risk group $u(E_i, C_t^{S_i})$. The expected future utility for exposed individuals is given by the potential future health state transitions weighted by their transition probabilities,

$$\begin{aligned} &\underbrace{(1 - P^{E_i})}_{\text{Prob. of remain exposed}} \underbrace{V_{t+1}(E_i)}_{\substack{\text{Future expected utility} \\ \text{being exposed}}} + \underbrace{P^{E_i}\sigma}_{\substack{\text{Prob. of becoming} \\ \text{asymptomatic}}} \underbrace{V_{t+1}(A_i)}_{\substack{\text{Future expected utility} \\ \text{being asymptomatic}}} \\ &+ \underbrace{P^{E_i}(1-\sigma)}_{\substack{\text{Prob. of becoming} \\ \text{symptomatic}}} \underbrace{V_{t+1}(I)}_{\substack{\text{Future expected utility} \\ \text{being symptomatic}}}. \end{aligned} \tag{C.4}$$

Notice again that in order to solve the expected utility of exposed individuals we need to formulate the Bellman's equations for the expected utility while being asymptomatic infectious, symptomatic, and recovered. The Bellman's equations for asymptomatic and symptomatic individuals are similar since they only depend on the probability of recovery. Following the previous formulations we get that,

$$V_t(I) = u(C_t^*) + \delta[(1 - P^{IR})V_{t+1}(I) + P^{IR}V_{t+1}(R)], \quad \text{(C.5)}$$

$$V_t(A_i) = u(C_t^{S_i}) + \delta[(1 - P^{A_iR})V_{t+1}(A_i) + P^{A_iR}V_{t+1}(R)], \quad \text{(C.6)}$$

where the immediate utility for asymptomatic individuals uses the risk-specific contact rate of susceptible individuals $u(C_t^{S_i})$, since we assume they are unaware of their health status. Finally, the Bellman's equation for recovered individuals accounts for the immediate and future expected utilities without health state transitions,

$$V_t(R) = u(C_t^*) + \delta V_{t+1}(R). \quad \text{(C.7)}$$

Optimal adaptive behavioral decisions

In this section we illustrate the dynamic programming method used to solve the Bellman's equations (2) − (8) of the main text via backward induction over the planning horizon $[t, t+\tau]$. By using *Bellman's principle of optimality*, the dynamic optimization problem over the whole planning horizon is divided into sequential subproblems over periods $[t, t+1]$, $[t+1, t+2]$,$\cdots$,$[t+\tau-1, t+\tau]$. We use the following boundary conditions to solve the dynamic programming problems, regardless of the individual's health status h:

- $V_{t+\tau+1}(h) = 0$, since it exceeds the boundary of the planning horizon $[t, t+\tau]$,
- $V_{t+\tau}(h) = u_{t+\tau}(h)$, since it is the last period of the planning horizon.

To find the optimal contact rate at the current time, we assume individuals evaluate the risk of infection over the planning horizon based on a constant projection of the system's current state.

Susceptible individual's optimal contact choice at time $t+\tau$: Since this is the last period of the planning horizon, we assume individuals only consider current benefits and exclude risk of infection and benefits beyond the current day. It follows that the optimal decision at $t+\tau$ is the contact rate maximizing the immediate utility,

$$V_{t+\tau}(S_i) = V_{t+\tau}(E_i) = u(C_{t+\tau}^*) \quad \text{(C.8)}$$

where $C_t^* = b/2$ and b stands for the maximum number of contacts. The value C^* is dependent on the shape of the utility function assumed. The utility function assumes a maximum number of contacts per time-step (b), it follows that the symmetric single peak utility function shape implies that the optimal contacts rate is $C^* = b/2$.

Susceptible individual's optimal contact choice at time $t+\tau-1$: To find the optimal contact rate at this period we should make use of the previously found optimal choice at time $t+\tau$. The optimization problem becomes

$$V_{t+\tau-1}(S_i) = \max_{C_{t+\tau-1}^{S_i}} \{u(C_{t+\tau-1}^{S_i}) + \delta[(1 - P^{S_iE_i})V_{t+\tau}(S_i) + P^{S_iE_i}(V_{t+\tau}(E_i))]\}, \quad \text{(C.9)}$$

$$= \max_{C_{t+\tau-1}^{S_i}} \{u(C_{t+\tau-1}^{S_i}) + \delta[(1 - P^{S_iE_i})u(C_{t+\tau}^*) + P^{S_iE_i}u(C_{t+\tau}^*)]\}, \quad \text{(C.10)}$$

therefore,

$$V_{t+\tau-1}(S_i) = \max_{C_{t+\tau-1}^{S_i}} \{u(C_t^{S_i}) + \delta[(1 - P^{S_iE_i})u(C_{t+\tau}^*) + P^{S_iE_i}u(C_{t+\tau}^*)]\} \quad \text{(C.11)}$$

where $P^{S_iE_i}$ are given by Eqns. B.6 and B.7. Finally, the optimal contact rate for the period $t+\tau-1$ can be obtained from Eqn. (C.11) by evaluating all the possible contact rates.

Susceptible individual's optimal contact choice at time $t+\tau-2$: To find the optimal contact rate at this period we make use of the optimal choices at $t+\tau$ and $t+\tau-1$. The optimization problem becomes,

$$V_{t+\tau-2}(S_i) = \max_{C^{S_i}_{t+\tau-2}} \{u(C^{S_i}_{t+\tau-2}) + \delta[(1-P^{S_iE_i})V_{t+\tau-1}(S_i) + P^{S_iE_i}V_{t+\tau-1}(E_i)]\}. \tag{C.12}$$

Notice that we require $V_{t+\tau-1}(E_i)$ to solve Eqn. (C.12). Making use of the Bellman's equation for exposed individuals at the period $t+\tau-1$, we get that,

$$V_{t+\tau-1}(E_i) = u(C^{S_i}_{t+\tau-1}) + \delta[(1-P^{E_i})V_{t+\tau}(E_i) + P^{E_i}(\sigma V_{t+\tau}(A_i) + (1-\sigma)V_{t+\tau}(I))], \tag{C.13}$$

$$= u(C^{S_i}_{t+\tau-1}) + \delta[(1-P^{E_i})u(C^*_{t+\tau}) + P^{E_i}(\sigma u(C^*_{t+\tau}) + (1-\sigma)u(C^*_{t+\tau})), \tag{C.14}$$

that is

$$V_{t+\tau-1}(E_i) = u(C^{S_i}_{t+\tau-1}) + \delta[(1-P^{E_i})u(C^*_{t+\tau}) + P^{E_i}\big(\sigma u(C^*_{t+\tau}) + (1-\sigma)u(C^*_{t+\tau})\big). \tag{C.15}$$

We replaced $u(C^{E_i}_{t+\tau-1})$ by $u(C^{S_i}_{t+\tau-1})$ we assume exposed individuals unaware of their health status behave as susceptible ones, therefore choosing the contact rate corresponding to the susceptible health state. Therefore, $V_{t+\tau-1}(E_i)$ is known, and Eqn. (C.12) can be solved by optimizing over all the possible contact rates.

Continuation of backward induction gives the optimal contact rates at each time during the planning horizon, particularly for the period $t+1$. Therefore, we use $C^{S_i}_{t+1}$ as the contact rate chosen by S_i susceptible individuals, and also chosen by exposed E_i and asymptomatic A_i individuals to run the epidemic model one period ahead. In Table C.1 we show the dependence of the expected utilities on the potential health-status during the optimization process

Pop.	$t+\tau-3$	$t+\tau-2$	$t+\tau-1$	$t+\tau$
S_i	S_i, E_i, A_i, I, R	S_i, E_i, A_i, I	S_i, E_i	S_i
E_i	E_i, A_i, I, R	E_i, A_i, I, R	E_i, A_i, I	E_i
A_i	A_i, R	A_i, R	A_i, R	A_i
I	I, R	I, R	I, R	I
R	R	R	R	R

Table C.1: Recursive dependence of the optimization problem

Figure C.1 shows a schematic of the coupling between the mean-field epidemic model and the model of adaptive human behavior.

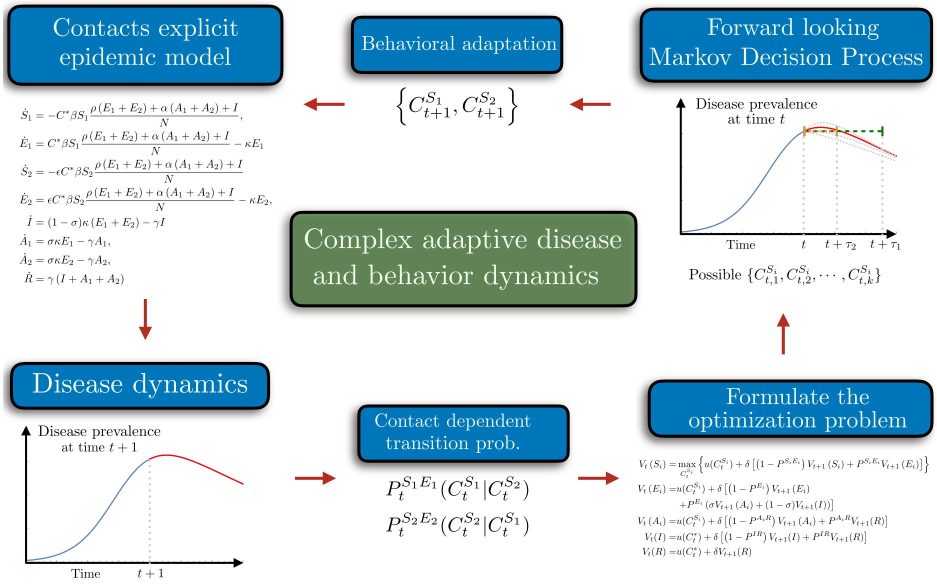

Figure C.1: **Forward looking adaptive behavior framework.** The epidemic and the behavioral models are coupled at each time step to generate a feedback loop: disease dynamics impact individual behavior and changes in individuals behavior modulate disease dynamics.

D Sensitivity analysis

Exposed individual infectiousness ρ

To the date, the infectiousness of pre-symptomatic individuals is still unknown. We assumed for our simulations a reduced infectious rate of exposed individuals, relative to symptomatic individuals, $\rho = 0.25$. In this section we study the impact of variations in the relative infectiousness of exposed individuals on the evolution of the epidemic and on the behavioral response produced.

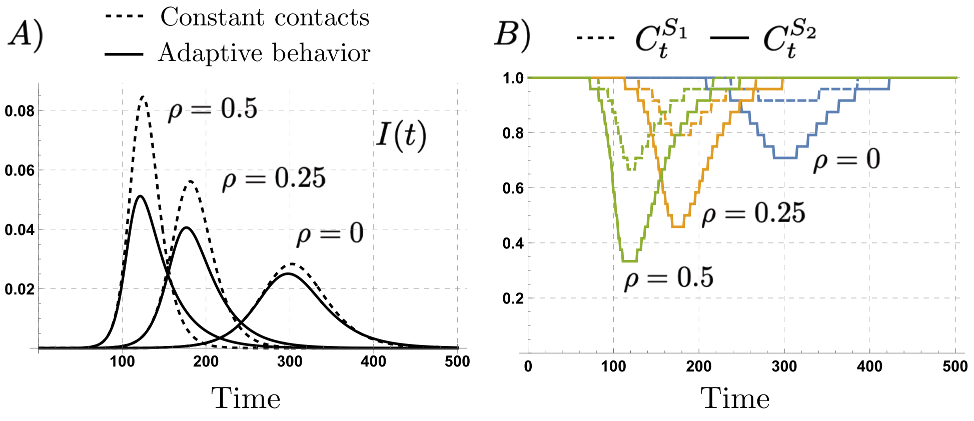

Figure D.1: Panel A: Disease dynamics under constant contact rates (dashed lines) and under adaptive behavior (solid lines). Panel B: Risk taker (dashed) and risk evader (solid) contact rates, $C_t^{S_1}$ and $C_t^{S_2}$, respectively. Exposed individuals' relative infectiousness of $\rho = 0, 0.25$ and 0.5, for the parameters set in Table (1). Increments on the exposed individuals' relative infectiousness increases the symptomatic class peak size and reduces the peak time. Moreover, the behavioral response of both risk groups increases.

The utility function $u(C) = \left(bC - C^2\right)^{\nu}$

The utility function is the cornerstone of our proposed behavioral model. However, in the absence of appropriate data to calibrate the group-specific behavioral responses, in this section we test the sensitivity of the behavioral responses and disease dynamics to changes in the utility parameters.

The maximum number of daily contacts (b)

We found the overall dynamics of the behavior model to have low sensitivity to the daily available contacts. For an assumed b value we calibrate the per-capita likelihood of infection β, so that the basic reproductive number of the behavior model matches the targeted basic reproductive value of $\mathcal{R}_0 = 2.4$.

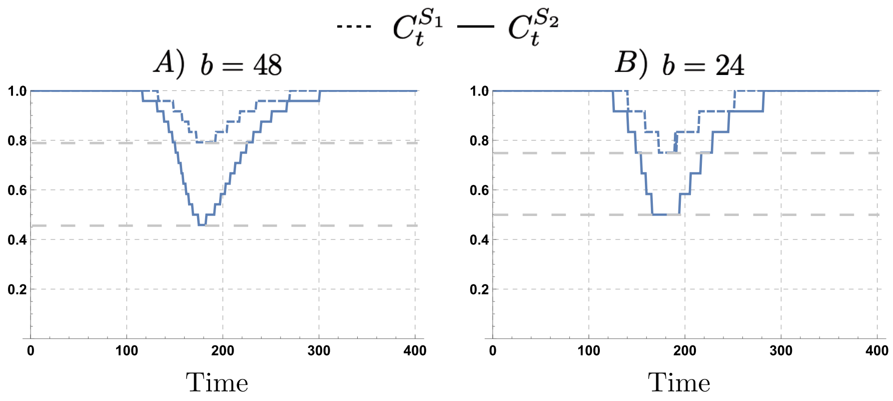

Figure D.2: Risk taker (dashed) and risk evader (solid) contact rates, $C_t^{S_1}$ and $C_t^{S_2}$, respectively. Exposed individuals' relative infectiousness of $b = 24$ and $b = 48$, for the parameters set in Table (1). We found our behavioral model to have low sensitivity to changes in the daily contact rates.

Figure D.2 shows the risk-group-specific adaptive contact rate for daily average contact rates of $b = 24$ and $b = 48$. We found that the behavioral responses slightly differ for different b values. Intuitively, the perceived difference is due to the refinement of the action space assumed for the Markov Decision Processes.

The utility function shape (ν)

In the proposed behavior model, adaptive response is triggered by assessing the benefits of making contacts, while being exposed to the risk of infection. The benefits-risks trade-off is ultimately dependent on the way individuals value their contacts. We use the ν parameter of the utility function to define the risk-group-specific marginal benefits of increasing contacts (the increment on utility obtained by increasing contacts $du(C)/dC$), ν_1 and ν_2. To study the system's sensitivity to changes in the risk-group-specific marginal benefits of increasing contacts, we focus on the impact of changes in ν_1 while fixing ν_2, and vice versa.

Figure D.3 shows different utility functions (panel A), and the associated marginal benefit/cost structures (panel B), for $\nu = 0.05, 0.1$ and 0.2. The immediate utility that individuals get by making C contacts is given by $u(C)$. The marginal benefits (costs) of increasing (decreasing) contacts, reduces as the ν value decreases. In other words, the utility function structure in terms of marginal benefits (losses) reflects individual dispositions to reduce their number of contacts during the epidemic.

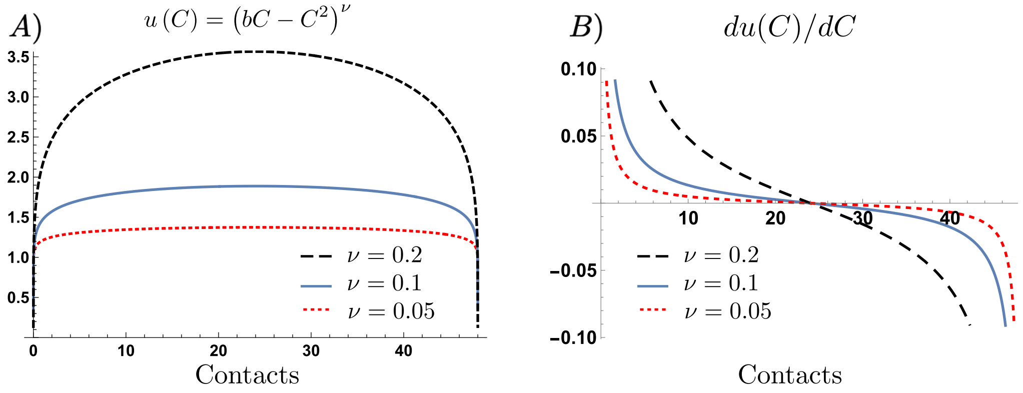

Figure D.3: Immediate utility of making C contacts (panel A), and the marginal benefit of increasing contacts (panel B), for ν values of 0.05, 0.1 and 0.2, and maximum daily contacts of $b = 48$.

We test the system's local sensitivity to changes of the risk-taker and risk-evader behavioral responses. Figures D.4 and D.5 show the epidemic dynamics of the symptomatic population, $I(t)$, and the risk-group-specific contact rates ($C_t^{S_1}$ and $C_t^{S_2}$), for varying ν_1 and ν_2 values. Notice that while the changes on the risk-taker contacts rate are directly given by modifying their contacts' marginal benefits/costs, risk evaders are

sensitive to these changes. In other words, changes in the contact rate of a given risk group modify the epidemic structure, which also impacts the behavioral responses of others.

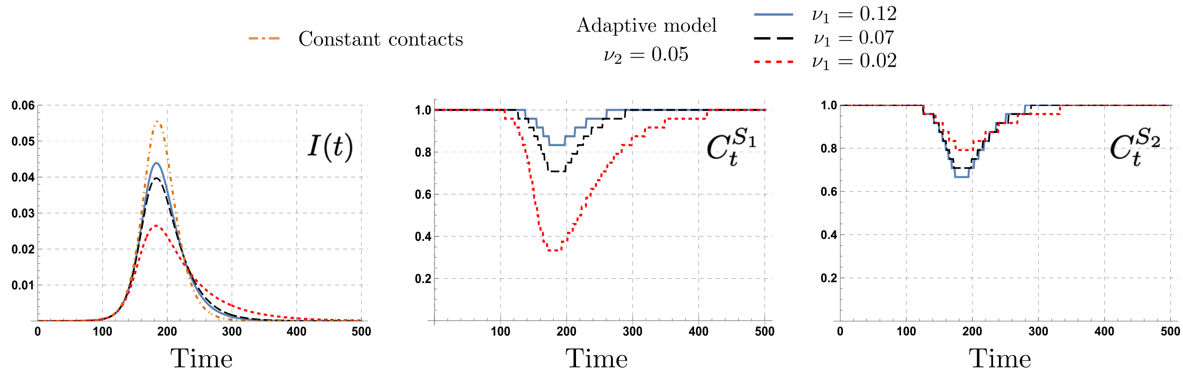

Figure D.4: Varying risk-taker utility structure ν_1 impact the progression of the epidemic by modulating both, the behavioral response of the risk-taker and the risk evader populations.

Figure D.5 shows the impact of varying the risk-evader utility structure for $\nu_2 = 0.02, 0.07$ and 0.12. By unilaterally varying the risk-evader utility function, the whole epidemic dynamics change due to a stronger/weaker behavioral response by this subpopulation. Moreover, our simulations show that the risk-taker behavioral responses are responsive but have low sensitivity to changes of the risk-evader contact rate.

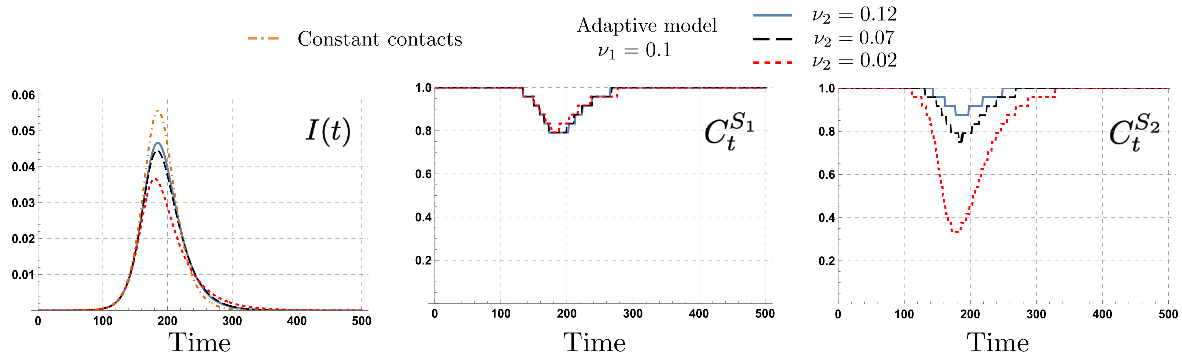

Figure D.5: Varying risk-evader utility structure ν_2 impact the progression of the epidemic by modulating the behavioral response of the risk-evader population, while the behavioral response of the risk-taker population shows almost no change.

References

[1] O. Diekmann, J. A. P. Heesterbeek, and J. A. J. Metz. On the definition and the computation of the basic reproduction ratio R_0 in models for infectious diseases in heterogeneous populations. *J. Math. Biol.*, 28(4):365–382, 1990.

[2] P. van den Driessche and J. Watmough. Reproduction numbers and sub-threshold endemic equilibria for compartmental models of disease transmission. *Math. Biosci.*, 180:29–48, 2002.
